# Gestational Age and Neurodevelopmental Delay in Term Births at 6 and 12 Months: The Japan Environment and Children’s Study (JECS)

**DOI:** 10.1007/s10995-024-03908-4

**Published:** 2024-03-11

**Authors:** Kentaro Haneda, Mitsuaki Hosoya, Keiya Fujimori, Seiji Yasumura, Hidekazu Nishigori, Masahito Kuse, Hyo Kyozuka, Hajime Maeda, Akiko Sato, Yuka Ogata, Koich Hashimoto, the Japan Environment and Children’s Study (JECS) Group

**Affiliations:** 1 Department of Pediatrics, School of Medicine, Fukushima Medical University, 1 Hikariga-oka, Fukushima, 960-1295 Japan; 2 Fukushima Regional Center for the Japan Environmental and Children’s Study, Fukushima, Japan; 3 Department of Obstetrics and Gynecology, School of Medicine, Fukushima Medical University, Fukushima, Japan; 4 Department of Public Health, School of Medicine, Fukushima Medical University, Fukushima, Japan; 5 Fukushima Medical Center for Children and Women, Fukushima Medical University, Fukushima, Japan

**Keywords:** Early term, Birth cohort, ASQ-3, Neurodevelopmental delay

## Abstract

**Background:**

In the recent years, a high risk of developmental delay not only in very low birth weight infants and late preterm infants but also in early term infants (37–38 weeks) have increasingly been reported. However, in Japan, there are virtually no studies regarding the development delays in early term infants.

**Methods:**

This study used the data from the Japan Environment and Children’s Study (JECS), a birth cohort study conducted in Japan. Data were selected for analysis from the records of 104,065 fetal records. The risk of neurodevelopmental delays at 6 months and 12 months after birth was evaluated using multivariate analysis for infants of various gestational ages, using the 40th week of pregnancy as a reference value. Neurodevelopment was evaluated at 6 months and 12 months after birth using the Ages and Stages Questionnaires, Japanese translation (J-ASQ-3).

**Results:**

The proportion of infants born at a gestational age of 37 to 38 weeks who did not reach the J-ASQ-3 score cutoff value was significantly higher in all areas at both 6 months and 12 months after birth, when compared to that of infants born at 40 weeks. The odds ratio decreased at 12 months after birth compared to that at 6 months after birth.

**Conclusion:**

Early term infants in Japan are at an increased risk of neurodevelopmental delay at 12 months after birth.

## Significance

**What’s known on this subject:**

Early term infants with gestational ages of 37 to 38 weeks and not only premature babies may be at an increased risk of developmental delay.

**What this study adds:**

This study was conducted using data from a birth cohort of 104,065 fetal records. Early term infants had an increased risk of developmental delay at 6 months, and while the risk decreased by 12 months after birth, it was still high.

## Introduction

Preterm birth is associated with developmental delays. However, in recent years an increasing number of studies have indicated a high risk of perinatal complications not only in very preterm and late preterm infants (34–36 weeks’ gestation) (Aakre et al., 2017; Allotey et al., 2018; Pascal et al., 2018), but also in early term infants (37–38 weeks) (Dong et al., 2012; Mahoney & Jain, 2013; Parikh et al., 2014). Furthermore, there is increasing evidence to suggest that early term infants may also be at higher risk of developmental delay, similar to preterm infants (MacKay et al., 2010; Noble et al., 2012; Wu et al., 2021).

Although it is known that the relationship between weeks of gestation and neurodevelopment in term infants can have an important influence on the timing of delivery, only two reports have assessed the risk by week of gestation within the range of term delivery(Espel et al., 2014; Gleason et al., 2022), both of which used the Bayley Scales of Infant Development Mental and Psychomotor exam. The Bayley Scale is a useful objective measure of mental and motor development in infants; however, it is difficult to apply widely because it is administered by clinicians. Nevertheless, early intervention for developmental delays remains an important consideration. Therefore, in the present study, we used data from the Japanese Environment and Children’s Study (JECS), which included the ASQ-3, a developmental screening test that can be easily administered by parents. Assessment of developmental delay in preterm infants using the ASQ-3 has been reported in prior studies (Demestre et al., 2016; Mirzakhani et al., 2020), and parental and professional assessments by the ASQ-3 are considered equivalent in outcome (Gutierrez-Cruz et al., 2019; Malak et al., 2022; Schonhaut et al., 2013). In the present study, we assessed neurodevelopment by gestational week in term babies born at 37–41 weeks’ gestation, by analysis of ASQ-3 scores at 6 and 12 months of age. Further, a subgroup analysis by gender was performed, as male premature infants are known to be at a higher risk of neurodevelopmental disorders (Johnson et al., 2015; Linsell et al., 2015; Lowe et al., 2019), while male early term infant have a reportedly higher risk of developmental delay (Wu et al., 2021).

## Participants and Methods

The data used in this study were obtained from the JECS population. The JECS is a nationwide, multicenter, prospective birth cohort study conducted by Ministry of the Environment of Japan. The objective of the JECS is to survey the relationship between growth/development from the prenatal period to infancy and childhood with the environment surrounding the child and the mother, particularly in regard to chemical exposure. This protocol has been previously reported (Kawamoto et al., 2014; Michikawa et al., 2018). The pregnant women who participated in this study were registered between January 2011 and March 2014 across 15 regions of Japan.

The protocol of this study was approved by the Ministry of the Environment’s Institutional Review Board on Epidemiological Studies, as well as by the ethics committees of all participating institutions. Consent was obtained from all participants via written notification. The JECS was conducted in accordance with the Declaration of Helsinki and other regulations and guidelines valid at the national level.

### Data Collection

This study was based on the jecs-an-20,180,131 dataset, released in March 2018. Data were collected from pregnancy until one year after delivery. Self-reported information on the mother’s socioeconomic status and preexisting medical conditions was obtained in the first and third trimesters of pregnancy via mailed questionnaires. Further, information on gestational weeks, obstetric outcomes, and neonatal information was also transcribed from the medical records at each participating institution. Infant neurodevelopmental assessments were also assessed by self-report received by mail at 6 and 12 months of age.

### Outcomes, Exposures, and Covariates

*The Ages & Stages Questionnaires* ® (ASQ-3™): *A Parent-Completed Child Monitoring System, Third edition* (Squires & Bricker, 2009) was employed for the evaluation of neural development. The ASQ-3 scores in infants at 6 and 12 months after birth was investigated as the primary outcome.

The ASQ-3 is a screening system conducted via a questionnaire answered by an infant’s guardian. This comprises 30 items related to development, which are divided into five categories: communication, gross motor, fine motor, problem solving, and personal-social. Guardians answer “yes,” “sometimes,” or “not yet” in response to all of the assessment items, with these answers assigned scores of 10, 5, and 0 points, respectively. The points scored in each development category are summed, and evaluation as to whether they surpass the cutoff values is performed (Squires, 2009). We use cut off values of the Japanese translation of the ASQ-3 (J‐ASQ‐3) (Mezawa et al., 2019). Values below the cutoff were deemed as “clinical” neural development abnormalities. Data from incomplete questionnaires were excluded. Responses were classified as incomplete if the age of the infant in months was ± 1 month off the 6- or 12-month time points, or if there were 3 or more unanswered items per category.

Patients with stillbirths or multiple births were excluded. Body abnormalities included not only major congenital anomalies, but also various minor malformations; however, all were excluded as it was difficult to determine the effect of each lesion described in the questionnaire on the neurological prognosis.

Gestational age was determined from either the last menstrual period, crown-rump length by ultrasound, or the date of artificial insemination/in vitro insemination. Cases were classified into five groups based on gestational age at birth: 37 weeks (including 37 weeks 0 days through to 37 weeks 6 days), 38, 39, 40, and 41 weeks.

We considered the following confounding factors to influence the data: sex, small for gestational age (SGA), Apgar score at 5 min, cesarean section, mother’s age at parturition, mother’s developmental disorders (Attention-Deficit Hyperactivity Disorder, Autism Spectrum Disorder/Pervasive Developmental Disorders, Learning Disability, and others), mother’s higher education, household income, and mother’s marital status. All data related to these variables were obtained from the JECS. The mother’s age at parturition was a continuous variable, while SGA, cesarean section, mother’s developmental disorders, mother’s higher education level, and mother’s marital status were binary variables classified as yes/no. SGA was defined as height and weight less than the 10th percentile at the time of birth, and was corrected by gestational age, sex, and primipara/multipara based on the “New Japanese neonatal anthropometric charts for gestational age at birth”(Itabashi et al., 2014). The educational status of the mother was classified as junior/senior high school or lower and higher than senior high school. The Apgar score at 5 minutes was classified into either < 7, or ≧ 7. Household income was classified as < 2 million yen, 2–6 million yen, 6–10 million yen, or > 10 million yen.

As males are at a higher risk of developmental delay due to preterm birth, we further stratified all participants by sex to perform a subgroup analysis to assess any potential differences between the ASQ-3 categories.

### Statistical Analysis

For univariate analysis between groups, Mann–Whitney U tests were used for continuous data, and chi-squared tests were applied for categorical data. Next, we investigated whether there was a significant association between the proportion of infants with “clinical” J-ASQ-3 scores in any of the developmental areas and gestational age, using a gestational age of 40 weeks as the reference value. Finally, we performed a subgroup analysis according to sex. Multiple logistic regression analysis was subsequently conducted to calculate the adjusted odds ratios (aOR) and 95% confidence intervals (CI). In two-sided tests, p values < 0.05 were considered statistically significant. SPSS 26.0 for Windows (SPSS Inc., Chicago, IL, USA) was used for all statistical analysis.

## Results

**Fig. 1:**
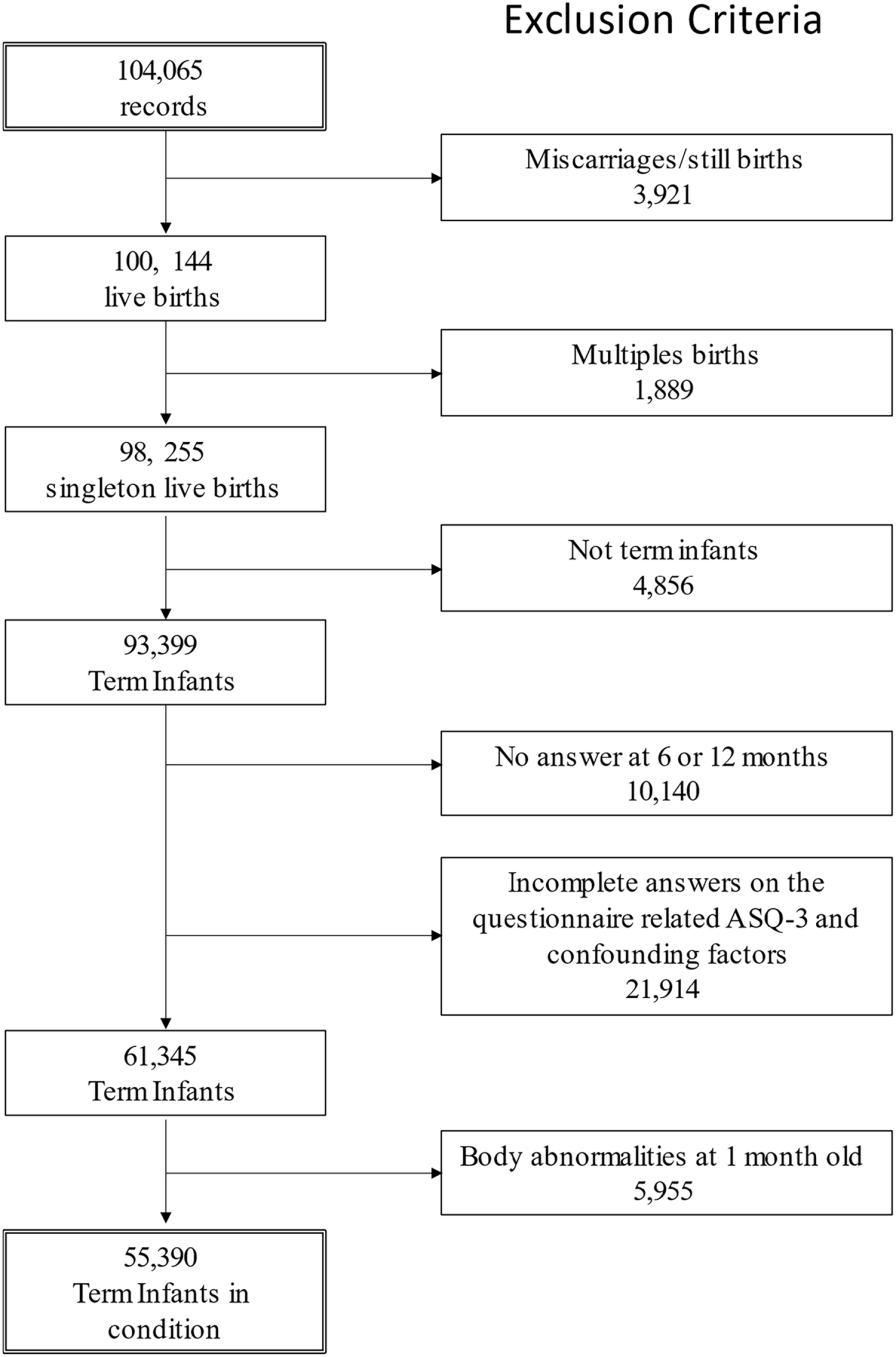
Flow chart of the infants analyzed from the Japan Environment and Children’s Study (JECS)

A total of 104,065 fetal records were registered in the JECS between 2011 and 2014. After applying our inclusion criteria, 55,390 participants were enrolled in the study (Fig. 1). The characteristics of children with normal scores for all developmental areas on the J-ASQ-3 at 6 and 12 months, as well as those with clinical scores for any developmental area are shown in Tables 1 and 2. In total, 11,481 (20.7%) and 7,461 (13.5%) children had clinical scores for any area at 6 and 12 months, respectively. SGA, low birth weight infants, infants with lower gestational age at birth and male had significantly higher clinical scores at 12 months, but this difference was not significant at 6 months. Moreover, infants of older mothers had significantly higher rates of not meeting the cutoff values. At 12 months after birth, the rate of infants whose ASQ-3 scores were below the cutoff in any area was 19.4% at 37 weeks, 16.5% at 38 weeks, and 10.9% at 40 weeks.

**Table 1:** Baseline characteristics of 55,390 singleton infants at 6 months

	Normal score at 6 months *n* = 43,907 (79.3)		Clinical scores for any area at 6 months *n* = 11,483(20.7)		p-value
Male sex	22,065	(50.3)	5825	(50.7)	0.186^a^
Gestational age at birth					
37weeks	3594	(8.2)	1733	(15.1)	< 0.001^b^
38weeks	9540	(21.7)	3382	(29.5)	
39weeks	13,195	(30.1)	3171	(27.6)	
40weeks	12,992	(29.6)	2487	(21.7)	
41weeks	4586	(10.4)	710	(6.2)	
Birthweight					
< 2,000	58	(0.1)	30	(0.3)	< 0.001^b^
2,000 ≦ to < 2,500	1934	(4.4)	815	(7.1)	
2,500 ≦ to < 3,000	16,617	(37.8)	4999	(43.5)	
3,000 ≦ to < 3,500	19,885	(45.3)	4625	(40.3)	
3,500≦	5413	(12.3)	1014	(8.8)	
SGA	1236	(2.8)	368	(3.2)	0.014^a^
Neonatal asphyxia of 5 min at birth	258	(0.6)	64	(0.6)	0.378^a^
Cesarean section	6979	(15.9)	2549	(22.2)	< 0.001^a^
Mother’s age					
< 20	210	(0.5)	29	(0.3)	< 0.001^b^
20–24	3558	(8.1)	526	(4.6)	
25–29	12,583	(28.7)	2702	(23.5)	
30–34	15,931	(36.3)	4385	(38.2)	
35–39	9713	(22.1)	3153	(27.5)	
>40	1912	(4.4)	688	(6.0)	

SGA, small for gestational age

Date are n(%) unless otherwise specified

^a^P-value, Chi-squared test

^b^P-value, Mann Whitney’s u test

Missing date n(%):

**Table 2:** Baseline characteristics of 55,390 singleton infants at 12 months

	Normal score at 12 months *n* = 47,927 (86.5)		Clinical score for any area at 12 months *n* = 7,463 (13.5)		p-value
Male sex	23,827	(49.7)	4063	(54.4)	< 0.001^a^
Gestational age at birth					
37weeks	4293	(9.0)	1.034	(13.9)	< 0.001^b^
38weeks	10,791	(22.5)	2131	(28.6)	
39weeks	14,317	(29.9)	2049	(27.4)	
40weeks	13,791	(28.8)	1688	(22.6)	
41weeks	4735	(9.9)	561	(7.5)	
Birthweight					
< 2,000	63	(0.1)	25	(0.3)	< 0.001^b^
2,000 ≦ to < 2,500	2253	(4.7)	496	(6.6)	
2,500 ≦ to < 3,000	18,451	(38.5)	3165	(42.4)	
3,000 ≦ to < 3,500	21,492	(44.8)	3,018	(40.4)	
3,500≦	5668	(11.8)	759	(10.2)	
SGA	1361	(2.8)	243	(3.3)	0.025^a^
Neonatal asphyxia of 5 min at birth	258	(0.5)	64	(0.9)	< 0.001^a^
Cesarean section	7841	(16.4)	1687	(22.6)	< 0.001^a^
Mother’s age					
< 20	226	(0.5)	13	(0.2)	< 0.001^b^
20–24	3743	(7.8)	341	(4.6)	
25–29	13,583	(28.3)	1702	(22.8)	
30–34	17,448	(36.4)	2868	(38.4)	
35–39	10,790	(22.5)	2076	(27.8)	
>40	2137	(4.5)	463	(6.2)	

SGA, small for gestational age

Date are n(%) unless otherwise specified

^a^P-value, Chi-squared test

^b^P-value, Mann-Whitney’s u test

Tables 3 and 4 show the gestational ages of infants and the adjusted odds ratios (aOR) obtained from the logistic analysis of the J-ASQ-3 subscale scores at 6 and 12 months after birth. The aOR of infants born at 37–38 weeks was higher than that of infants born at 40 weeks at both 6 and 12 months [aOR at 6 months (communication:1.74, gross motor:2.28, fine motor:2.45, problem solving:2.48, personal-social:3.27 (37 weeks); 1.21, 1.29, 1.38, 1.34, 1.46 (38 weeks)), at 12 months (2.82, 1.72, 1.78, 1.49, 2.68 (37 weeks); 1.76, 1.18, 1.21, 1.22, 1.46 (38 weeks))]. Only the communication subscale scores at 37 weeks and 12 months showed no significant differences. In addition, compared to 40 weeks, a gestational age of either 39 or 41 weeks had either no effect, or an extremely small effect. Overall, the effect of gestational age was greater on the communication and personal-social subscales.

**Table 3:** Relation between gestational age and below cut off by J-ASQ-3 at 6 months

Developmental area						
Gestational age		Communication	Gross motor	Fine motor	Problem solving	Personal-social
41wks						
aOR (95%Cl)		1.06(0.95–1.18)	0.97(0.94–0.99)	0.97(0.93–1.01)	0.93(0.90–0.96)	0.97(0.91–1.02)
40wks						
aOR (95%Cl)		Ref.	Ref.	Ref.	Ref.	Ref.
39wks						
aOR (95%Cl)		1.01(0.91–1.13)	1.08(1.05–1.11)	1.10(1.06–1.14)	1.08(1.05–1.10)	1.11(1.06–1.16)
38wks						
aOR (95%Cl)		1.21(1.04–1.41)	1.29(1.24–1.35)	1.38(1.31–1.46)	1.34(1.29–1.40)	1.46(1.37–1.56)
37wks						
aOR (95%Cl)		1.74(1.19–2.52)	2.28(2.08–2.51)	2.43(2.13–2.77)	2.48(2.27–2.72)	3.27(2.82–3.80)

aOR, adjusted Odds Ratio:  CI Confidence interval; Ref Reference

Adjusted for sex, small for gestational age (SGA), Apgar score at 5 min, cesarean section, age of the mother at parturition, mother’s developmental disorders, mother’s higher education, household income, and mother’s marital status

**Table 4:** Relation between gestational age and below cut off of J-ASQ-3 at 12 months

Developmental area						
Gestational age		Communication	Gross motor	Fine motor	Problem solving	Personal-social
41wks						
aOR (95%Cl)		1.12(0.80–1.59)	1.00(0.96–1.03)	0.98(0.94–1.02)	1.00(0.96–1.04)	0.94(0.85–1.05)
40wks						
aOR (95%Cl)		Ref.	Ref.	Ref.	Ref.	Ref.
39wks						
aOR (95%Cl)		1.34(0.98–1.84)	1.03(0.99–1.07)	1.04(1.00-1.07)	1.07(1.03–1.10)	1.17(1.08–1.26)
38wks						
aOR (95%Cl)		1.76(1.10–2.81)	1.18(1.11–1.25)	1.21(1.15–1.28)	1.22(1.15–1.28)	1.46(1.30–1.64)
37wks						
aOR (95%Cl)		2.82(0.91–8.75)	1.72(1.49–1.99)	1.78(1.57–2.02)	1.49(1.27–1.73)	2.68(2.04–3.52)

aOR, adjusted Odds Ratio: CI, CI,confidence interval; Ref, reference

Adjusted for sex, small for gestational age (SGA), Apgar score at 5 min, cesarean section, age of the mother at parturition, mother’s developmental disorders, mother’s higher education, household income, and mother’s marital status

Tables 5 and 6 present the results of the subgroup analyses of sex at 6 and 12 months, respectively. There was sex difference only in communication area at 6 months of age.　 However, there was few sex difference in either area at 12 months of age.

**Table 5:** Relation between gestational age and below cut off by J-ASQ-3 at 6 months by sex

Developmental area											
Gestational age		Communication		Gross motor		Fine motor		Problem solving		Personal-social	
		M	F	M	F	M	F	M	F	M	F
41wks											
aOR (95%Cl)		1.13 (0.98–1.32)	0.99 (0.85–1.16)	0.99 (0.95–1.04)	0.95 (0.91–0.99)	0.99 (0.93–1.06)	0.95 (0.90–1.01)	0.94 (0.89–0.98)	0.92 (0.88–0.96)	0.95 (0.88–1.03)	0.98 (0.91–1.06)
40wks											
aOR (95%Cl)		Ref.	Ref.	Ref.	Ref.	Ref.	Ref.	Ref.	Ref.	Ref.	Ref.
39wks											
aOR (95%Cl)		1.01 (0.86–1.18)	1.02 (0.89–1.18)	1.11 (1.06–1.15)	1.06 (1.02–1.10)	1.10 (1.05–1.17)	1.09 (1.03–1.15)	1.07 (1.03–1.11)	1.08 (1.04–1.12)	1.13 (1.06–1.20)	1.07 (0.99–1.15)
38wks											
aOR (95%Cl)		1.36 (1.09–1.69)	1.07 (0.85–1.33)	1.36 (1.28–1.44)	1.24 (1.17–1.31)	1.42 (1.31–1.54)	1.35 (1.25–1.46)	1.34 (1.26–1.41)	1.36 (1.28–1.43)	1.47 (1.35–1.61)	1.45 (1.31–1.60)
37wks											
aOR (95%Cl)		1.79 (1.04–3.09)	1.74 (1.04–2.92)	2.47 (2.16–2.82)	2.11 (1.84–2.41)	2.48 (2.06–2.98)	2.40 (2.00-2.88)	2.31 (2.03–2.63)	2.70 (2.37–3.07)	3.34 (2.75–4.07)	3.15 (2.51–3.94)

M, male; F, female; aOR, adjusted Odds Ratio: CI, CI,confidence interval; Ref, reference

Adjusted for small for gestational age (SGA), Apgar score at 5 min, cesarean section, age of the mother, mother’s developmental disorders, mother’s higher education, household income, and mother’s marital status

**Table 6:** Relation between gestational age and below cut off by J-ASQ-3 at 12 months by sex

Developmental area											
Gestational age		Communication		Gross motor		Fine motor		Problem solving		Personal-social	
		M	F	M	F	M	F	M	F	M	F
41wks											
aOR (95%Cl)		1.03 (0.59–1.82)	1.19 (0.76–1.86)	0.99 (0.93–1.05)	1.00 (0.95–1.05)	0.97 (0.92–1.02)	0.99 (0.93–1.05)	1.00 (0.95–1.05)	1.00 (0.94–1.06)	0.91 (0.78–1.06)	0.98 (0.85–1.12)
40wks											
aOR (95%Cl)		Ref.	Ref.	Ref.	Ref.	Ref.	Ref.	Ref.	Ref.	Ref.	Ref.
39wks											
aOR (95%Cl)		1.51 (0.99–2.31)	1.11 (0.67–1.82)	1.03 (0.97–1.08)	1.03 (0.99–1.08)	1.02 (0.98–1.07)	1.06 (1.01–1.12)	1.05 (1.00-1.10)	1.09 (1.03–1.15)	1.21 (1.08–1.34)	1.13 (1.00-1.27)
38wks											
aOR (95%Cl)		1.87 (0.99–3.55)	1.63 (0.81–3.26)	1.16 (1.06–1.25)	1.20 (1.11–1.29)	1.16 (1.08–1.24)	1.30 (1.19–1.42)	1.19 (1.11–1.28)	1.25 (1.15–1.36)	1.55 (1.33–1.81)	1.35 (1.13–1.61)
37wks											
aOR (95%Cl)		3.29 (0.73–14.7)	4.26 (0.71–25.4)	1.86 (1.55–2.24)	1.71 (1.39–2.10)	1.62 (1.38–1.90)	2.07 (1.70–2.53)	1.51 (1.27–1.80)	1.59 (1.24–2.04)	2.76 (1.92-4.00)	2.42 (1.59–3.69)

M male; F female; aOR adjusted Odds Ratio; CI Confidence interval; Ref reference

Adjusted for small for gestational age (SGA), Apgar score at 5 min, cesarean section, age of the mother, mother’s developmental disorders, mother’s higher education, household income, and mother’s marital status

## Discussion

In our study, early term infants were at a higher risk of developmental delay. Preterm infants, including late preterm infants, were followed carefully as they are known to be at higher risk of developmental delay; however, the proportion of infants from the JECS born at less than 37 weeks’ gestation was only 4.5% (Takami et al., 2018). Conversely, 31.8% of the cohort in this study comprised early term infants born at 37–38 weeks. This means that early term infants represent a very large proportion of all infants at risk of developmental delay. However, this population has often been overlooked in the past.

In a study on developmental delay in preterm infants, Gleason et al.(Gleason et al., 2022) assessed neurocognitive performance at 8, 4, and 7 years of age. They found that at 8 months, neurocognitive function improved as the gestational age approached 40 weeks. These results are similar to our findings at six months of age. However, this prior study states that the results at 7 years were similar, which differs slightly from our results at 12 months; although this difference could well be explained by the large disparity in assessment points between the two studies. Chen et al.(Chen et al., 2022) also reported that only the delay in fine motor neurodevelopment showed a significant different at 6 months. Nevertheless, these reports and the results of our study all suggest that early term infants are at a higher risk of developmental delay.

According to the Infant Nutrition Survey by the Ministry of Health, Labor and Welfare in Japan, the proportion of infants born at gestational ages of 36–37 weeks increased from 11.7% in 2005 to 14.3% in 2015 (“Ministry of Health LaW. Nutrition Examination Survey of infants in 2005. https://www.e-stat.go.jp/stat-search/files?tstat=000001024531&toukei=00450271&cycle=8&tclass1=000001087457&layout=datalist&page=1&second2=1. Accessed 2/19, 2019.,” ; “Ministry of Health LaW. Nutrition Examination Survey of infants in (2015). https://www.e-stat.go.jp/stat-search/files?tstat=000001024531&toukei=00450271&cycle=8&tclass1=000001105135&tclass2=000001105136&layout=datalist&page=1&second2=1. Accessed 2/19, 2019“). If, as was shown in our study, early term infants are at an increased risk of developmental delay, the number of developmentally delayed children may increase as the number of early term infants increases. In this study, early term infants had significantly lower J-ASQ-3 scores at 6 months after birth. Early term infants may be found to have developmental delays in routine childhood health checks, which can cause anxiety in parents. In contrast, the aOR at 12 months after birth was lower than that at 6 months in this study. This may be because the risk of developmental delays associated with gestational age is reversible and could be influence by many factors. In other words, the effects of gestational age on early term infants can be reduced by the implementation of appropriate intervention from early infancy, and such interventions may be connected to building a better child-rearing environment. The results of the study by Gleason et al. were based on infants born more than 50 years ago, and it is likely that the children’s environment was different from that today. The differences between the results of our study and those of previous studies support this hypothesis. In support of our findings, one Chinese cohort study of 4243 newborns, including 1288 early term infants, also described the importance of early intervention for high-risk infants to improve neurodevelopmental outcomes (Stephenson et al., 2023). Overall, these results suggest that the J-ASQ-3, a parental screening tool, may help to more easily detect early developmental delay in early term infants.

In this study, 37.4% of the newborns at gestational ages of 37–38 weeks were born via cesarean section. In contrast, less than 6.0% of newborns at gestational ages of 39–40 weeks in the same dataset were born via cesarean section. Many cohort studies have reported an increase in perinatal complications such as respiratory disorders, neonatal sepsis, and low blood sugar in early term infants born after 37 or 38 weeks of pregnancy (Sengupta et al., 2013; Tita et al., 2009; Wilmink et al., 2010). In the US and the UK, cesarean sections are recommended after 39 weeks of pregnancy (American College of & Gynecologists, 2013; Health, 2011). Conversely, the Japanese Society of Obstetrics and Gynecology guidelines state that there is a low frequency of respiratory disorders in Japan in infants born by elective cesarean section at 38 weeks, and that the increase in emergency and after-hours cesarean sections may increase the risks to mothers and their infants. This suggests that elective cesarean section is a valid option at 38 weeks of pregnancy. In addition, the guidelines state that elective cesarean section at 37 weeks of pregnancy is a possible option, considering both the benefits and risk of the onset of respiratory disorders in infants. However, our data showed that in Japan, an extended period of pregnancy may decrease the risk of developmental delay. Emma et al. previously showed that elective caesarean sections should be avoided before 39 weeks, even in full-term babies, due to the impact of the week of conception on neurological and motor function (Espel et al., 2014). Unfortunately, the dataset used in our study did not indicate whether cesarean sections were elective. Because different countries show wide disparities in medical systems, they cannot be compared. As such, there is still a need to investigate the long-term effects of delaying planned births, not just the short-term effects, in Japan.

Premature infants are generally considered to be at high risk of developing developmental disorders, with gender also being an important factor. In our study, we observed a small sex gap among term infants, while infants born at 37weeks had bigger sex difference in the aOR. More data on late preterm infants are required to clarify these results.

This study has a few limitations that should be noted. First, the data analysis was limited to 12 months of age after birth; therefore, the long-term effects of exact gestational age are unclear. Furthermore, it is well known that developmental delays are caused by multiple factors, including both environmental and genetic factors; therefore, as an infant gets older, the effects of gestational age may decrease. Secondly, the J-ASQ‐3 cut‐off scores we used were calculated using data collected from participants living in a limited range of geographical locations in Japan. The score distribution derived in this study may not be representative of the score distribution among Japanese children in other parts of the country. However, similar results were obtained in the analysis of the ASQ-3, with results showing that infants born at 37–38 weeks had a high aOR. Finally, it is possible that we did not eliminate all other confounding factors.

## Conclusion

In conclusion, our analyses using the ASQ-3 revealed a relationship between lower gestational age and an increased risk of developmental delays at 6 and 12 months after birth in a large-scale cohort of term infants. A follow-up study has been planned which will collect when the infants reach 3 years old, to investigate the relationship between gestational age and neuromotor development in children.

## Data Availability

Data are unsuitable for public deposition due to ethical restrictions and legal framework of Japan. It is prohibited by the Act on the Protection of Personal Information (Act No. 57 of 30 May 2003, amendment on 9 September 2015) to publicly deposit the data containing personal information. Ethical Guidelines for Medical and Health Research Involving Human Subjects enforced by the Japan Ministry of Education, Culture, Sports, Science and Technology and the Ministry of Health, Labour and Welfare also restricts the open sharing of the epidemiologic data. All inquiries about access to data should be sent to: jecs-en@nies.go.jp. The person responsible for handling enquiries sent to this e-mail address is Dr Shoji F. Nakayama, JECS Programme Office, National Institute for Environmental Studies.
